# Bone marrow fibrosis in chronic myelomonocytic leukemia is associated with increased megakaryopoiesis, splenomegaly and with a shorter median time to disease progression

**DOI:** 10.18632/oncotarget.21870

**Published:** 2017-10-17

**Authors:** Kseniya Petrova-Drus, April Chiu, Elizabeth Margolskee, Sharon Barouk-Fox, Julia Geyer, Ahmet Dogan, Attilio Orazi

**Affiliations:** ^1^ Department of Pathology, Memorial Sloan Kettering Cancer Center, New York, NY, USA; ^2^ Department of Laboratory Medicine and Pathology, Mayo Clinic, Rochester, MN, USA; ^3^ Department of Pathology and Laboratory Medicine, New York Presbyterian Hospital–Weill Cornell Medicine, New York, NY, USA

**Keywords:** chronic myelomonocytic leukemia, bone marrow fibrosis, myeloid neoplasms

## Abstract

Bone marrow (BM) fibrosis is an adverse prognostic marker in several myeloid neoplasms, particularly in myelodysplastic syndrome (MDS) with fibrosis; however, its significance in chronic myelomonoctyic leukemia (CMML) has not been evaluated. We performed a retrospective analysis to investigate the prognostic and clinicopathological features of CMML with and without BM fibrosis. The study included specimens from a total of 83 untreated CMML patients from 2 large institutions. Patients with any amount of BM fibrosis (MF-1 or higher; MF1+) had significantly shorter progression-free survival (MF1+, 28.3 months vs MF0, not reached; *p* = 0.001, log rank test), splenomegaly (*p* = 0.016), and increased BM megakaryocytes (*p* = 0.04) compared to patients without BM fibrosis (MF-0). No association was observed between fibrosis and peripheral blood parameters, presence of JAK2 V617F mutation, BM blasts, or overall survival. Our study demonstrates the importance of assessing BM fibrosis in CMML. Similar to MDS, the presence of BM fibrosis may identify a distinct subgroup of CMML patients (CMML-F) with a more aggressive clinical course.

## INTRODUCTION

Chronic myelomonocytic leukemia (CMML) is a clonal hematopoietic stem cell disorder with overlapping myelodysplastic and myeloproliferative features, characterized by persistent peripheral blood (PB) monocytosis (≥ 1 × 10^9^/L), with monocytes comprising ≥ 10% of the PB leukocytes, and the presence of dysplasia in one or more hematopoietic lineages [1]. Since the original recognition of this neoplasm by the French-American-British (FAB) cooperative effort in 1982 [2], CMML has undergone several revisions and has been recognized by the 2001 [3] and 2008 [1] WHO Classifications as part of the overlap group of myelodysplastic/ myeloproliferative neoplasms (MDS/MPN). CMML is a clinically heterogeneous malignancy with variable risk of progression to acute myeloid leukemia (AML) and wide differences in survival with median ranges of 20–40 months [4]. In an effort to help stratify individual patient risk, the 2008 WHO classification recognized 2 blast-based categories: CMML-1 with < 5% blasts and blast equivalents (i.e. promonocytes) in the PB and < 10% in the BM, and CMML-2 where these account for 5–19% and 10–19%, respectively, or if there is presence of Auer rods [1]. In an effort to improve prognostication, the 2016 revision of the WHO Classification introduced a 3rd category (CMML-0) for cases with < 2% blasts in the PB and < 5% in the BM [5]. The FAB classification utilized degree of leukocytosis to identify two prognostic subgroups [2] namely “proliferative” (WBC count ≥ 13 X 10^9^/L) and “dysplastic” (WBC count < 13 × 10^9^/L) sub-types of CMML. Although this stratification fell to disuse over the years, based on clinical and recently discovered molecular differences in signaling pathways, the 2016 WHO revision re-recognizes these two subgroups [5].

In an effort to better predict patient outcomes in this heterogeneous disease, different prognostication systems have been introduced in the last three decades [6–10] evaluating a range of clinical, morphologic, and laboratory parameters; including hemoglobin levels, transfusion dependence, degree of leukocytosis, BM blast counts, and cytogenetic abnormalities, among others. While no specific karyotypic abnormalities have been associated with CMML, a number of somatic mutations have been identified in > 90% of CMML patients [11, 12]. Mutations in *TET2* and *SRSF2* genes are the most prevalent in CMML, and have been found to be specific for myeloid neoplasms with monocytosis [13]. Recently, several prognostication tools have emerged that incorporate molecular findings [14, 15]. The clinical/ molecular CMML-specific prognostic scoring system (CPSS-Mol) has improved risk stratification [16] by integrating the mutational status of certain genes, especially *ASXL1*, *NRAS*, *RUNX1*, and *SETBP1*, which are associated with poorer prognosis. The heterogeneity of CMML is further highlighted by the large differences in survival reported in this study with median survival of 18 months in the highest risk group and > 144 months in the lowest risk cohort.

Evaluation of BM morphology is an important part of the diagnostic assessment in myeloid neoplasms, and bone marrow biopsy is particularly invaluable to assess the presence of BM fibrosis [17]. Mild to moderate increase in reticulin fibrosis can be seen in nearly 30% of patients with CMML [18]. Evaluation of BM fibrosis is important for both diagnostic and prognostic assessment in a number of myeloid neoplasms, and a 2005 European consensus proposal for the semi-quantitative grading of BM fibrosis has helped to minimize inter-observer variability [19]. In MPN, including primary myelofibrosis (PMF) [20], essential thrombocythemia (ET)[21], and polycythemia vera (PV) [22], increased BM fibrosis is associated with poor prognosis. Studies have shown that fibrosis in primary MDS and therapy-related MDS correlates with worse survival [23–25]. Furthermore, incorporating information on BM fibrosis into risk stratification of MDS has been shown to be valuable in both retrospective [26] and prospective studies [27]. While the presence of BM reticulin fibrosis is seen in a proportion of CMML cases, its prognostic relevance has not been examined. The aim of this retrospective study was to compare the clinicopathologic features of CMML with and without BM fibrosis and to assess its prognostic value.

## RESULTS

Overall, 83 patients fulfilled the study criteria, and included 32 (39%) women and 51 (61%) men, with an average age of 68 years (range 23–90). Presence of BM fibrosis (grade MF-1 and above, MF1+) was identified in 45 (54%) patients, while 38 (46%) had no fibrosis (MF-0) at the time of initial evaluation. Patients with BM fibrosis were considered as a group, and included MF-1 (31; 37%), MF-2 (12; 14%), and MF-3 (2; 2%). When comparing MF-0 to MF1+, there were no differences with relation to age (mean: 70 vs 67 years, *p* = 0.1), gender (61% vs 62% men, *p* = 1), or blast-based CMML subtype (CMML-1, 86% vs 91%; CMML-2, 14% vs 9%, *p* = 0.5) (Table 1).

**Table 1:** Comparison of diagnostic categories, splenomegaly, treatment, and disease progression in patients without BM fibrosis to those with any amount of BM fibrosis

	MF-0 (*N* = 38)		MF1+ (*N* = 45)		*P*
**Mean Age (Range)**	70.1	41–90	66.6	23–87	0.1
**M**	23	61%	28	62%	1
**F**	15	39%	17	38%	
**CMML Category**					
1	32	86%	39	91%	0.5
2	5	14%	4	9%	
**CMML Type**					
MPN-type	20	53%	27	60%	0.6
MDS-type	18	47%	18	40%	
**Splenomegaly**					
Y	11/38	29%	26/45	58%	0.016
**Disease Progression**	6/33	18%	22/44	50%	0.005
**Median Time to Disease Progression (m)**	NR		28.3		0.001
**Median overall survival (m)**	48.3		41.7		0.1
**Treatment**					
Observation	10/33	31%	11/44	25%	0.95
Hypomethylating agents	13/33	39%	19/44	43%	
JAK inhibitor	1/33	3%	1/44	2%	
SCT	9/33	27%	13/44	30%	
**Outcome**					
DOD	9	24%	21	47%	0.6
ANED or AWD	12	32%	7	16%	
LFU or DUC or DUD	17	45%	17	38%	

NR = not reached, SCT = stem cell transplant, DOD = died of disease, ANED = alive no evidence of disease, AWD = alive with disease, DUC = died of unknown cause, DUD = died of unrelated disease, LFU = lost to follow up.

### Comparison of morphologic and molecular findings

No difference was observed between the MF-0 and MF1+ groups with respect to the WBC-based CMML subtype (proliferative-type: 53% vs 60%, dysplastic-type: 47% vs 40%; *p* = 0.6). The incidence of anemia, thrombocytopenia, MCV values, absolute monocyte count, and circulating peripheral blood blasts were also similar (Table 2). The MF1+ group showed a trend toward higher WBC count compared to MF-0; however, the difference was not statistically significant (mean 29.5 K/uL vs 19.5 K/uL; *p* = 0.06) (Table 2).

**Table 2:** Comparison of BM, PB, cytogenetic, and molecular findings in patients without BM fibrosis to those with any amount of BM fibrosis

	MF-0 (*N* = 38)		MF1+ (*N* = 45)		*P*
**Mean BM Cellularity (Range)**	80	(40–100)	85	(40–100)	0.1
**BM Megakaryocytes**					
Increased	17	45%	27	60%	0.04
Decreased	3	8%	8	18%	
Adequate	18	47%	10	22%	
**Mean BM Smear Blasts (Range)**	8.40	(1–15)	8.70	(1–16)	
**Blasts estimated by IHC (Number of cases**)	1/38	3%	4/45	9%	0.2
**Mean BM Smear Monocytes (Range)**	18	(3–34)	18	(2–35)	0.9
**Means (Range)**					
**WBC [x 10**^9^ **cells/ L]**	19.5	(3.7–53.1)	29.5	(4.1–170.2)	0.06
**PB Blasts (%)**	0.4	(0–8)	0.9	(0–7)	0.07
**Abs Mono [x 10**^9^ **cells/ L]**	4,806.2	(909–22833)	9,881.6	(988–131054)	0.1
**Hgb [g/ dL]**	10.8	(5–14.9)	10.8	(6.4–15)	0.9
**MCV [fL]**	91.5	(68.7–114)	91.8	(74–117)	0.9
**Plt [x10**^9^ **cells/ L]**	122	(32–296)	107.5	(8–504)	0.4
**Cytogenetic risk stratification**	*N* = 37		*N* = 43		0.8
low (normal karyotype, isolated -Y)	31	84%	34	79%	
intermediate (all other abnormalities)	4	11%	5	12%	
high (complex karyotype, +8, abnormalities of 7)	2	5%	4	9%	
**Molecular findings**					
*JAK2* V617F status	*N* = 27		*N* = 35		
negative	23	85%	33	94%	0.38
positive	4	15%	2	6%	
not tested	11		10		
*MPL* exon 10 mutation	*N* = 13		*N* = 15		
negative	13		15		1
positive	0		0		
not tested	25		30		
*CALR* exon 9 mutation	*N* = 5		*N* = 2		
negative	5		2		1
positive	0		0		
not tested	33		43		

Comparison of the BM parameters showed a statistically significant higher frequency of increased megakaryocytes in the MF1+ group (MF-0, 45% vs MF1+, 60%; *p* = 0.04), while no difference was seen in BM cellularity (MF-0, 80% vs MF1+, 85%; *p* = 0.1) (Table 2). BM blast and monocyte percentages were similar between these two groups (Table 2). In addition, blast enumeration was limited due to inadequate aspirate smears in a similar proportion of cases between the two groups, requiring estimation based on biopsy immunohistology (Table 2). The status of *JAK2* V617F mutation was available for a subset of the patients (27/38 in MF-0 and 35/45 in MF1+) at the time of the diagnostic assessment, the presence of which was similar in both groups [4/27 (15%) MF-0 vs 2/35 (6%) MF1+, *p* = 0.4] (Table 2). We did not appreciate any BM morphologic features of PMF among patients that are positive for a *JAK2* V617F mutation, akin to the CMML-like morphology seen in patients with PMF whose disease progression is signaled by monocytosis [28]. Furthermore, no specific megakaryocytic morphologic features with respect to nuclear lobation and hyperchromasia, size, and clustering were observed in patients with and without fibrosis. There were no mutations in *MPL* exon 10 (tested in 28 patients) or *CALR* exon 9 (tested in 7 patients). Karyotype analysis concurrent to the initial diagnosis similarly showed no statistically significant differences with regard to cytogenetic risk stratification between the two groups (Table 2).

### Comparison of clinical parameters and follow-up

**Figure 1:**
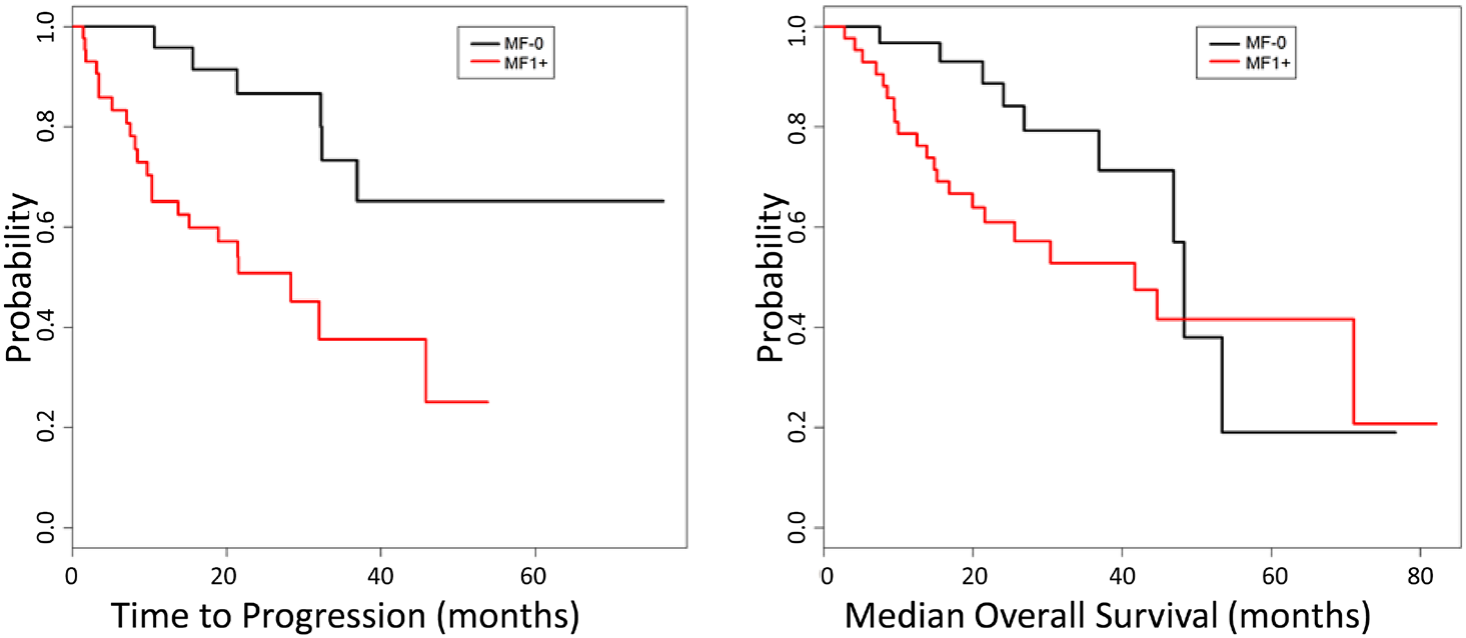
Kaplan-Meier analysis of disease progression CMML patients with MF1+ showed a shorter progression-free survival (28.3 months vs not reached, *p* = 0.001 log rank test) compared to MF-0 patients (left). No difference was observed in the median overall survival (MF-0: 48.3 vs MF1+:41,7 months, *p* = 0.1) between these two groups (right).

CMML patients with MF1+ showed a higher frequency of splenomegaly (MF1+, 58% vs MF0, 29%; *p* = 0.016) (Table 1). Follow-up information, including subsequent BM and PB assessment to evaluate for disease progression to a higher blast-based CMML category, AML, or myeloid sarcoma, was available for 44/45 (98%) of MF1+ and 33/38 (87%) of MF-0 groups. Patients in the MF1+ group were significantly more likely to experience disease progression when compared to MF-0 (22/44 (50%) vs 6/33 (18%); *p* = 0.005, Table 1) and a shorter median time to disease progression by Kaplan-Meier survival analysis (MF1+, 28.3 months vs MF0, not reached; *p* = 0.001, log rank test, Figure 1). The frequency of watchful waiting, treatment with hypomethylating agents, JAK inhibitor, hematopoietic stem cell transplant, the median overall survival, and the fraction of patients that died of their disease was similar between these two groups (Table 1). Comparison was also performed between MF 0–1 vs MF 2-3 groups; however, no significance was observed likely due to the small number of patients in the more fibrotic categories, limiting statistical power to detect a difference (69 vs 14 patients compared).

## DISCUSSION

Presence of BM fibrosis has been shown to be associated with more aggressive disease in a number of myeloid neoplasms, including MDS, ET, and PV [21–26]. While the diagnostic guidelines for CMML suggest assessment of BM fibrosis [1], prior studies have not evaluated either its prognostic significance or clinicopathological correlates. In this study, we find that assessment of the BM biopsy for fibrosis with a reticulin stain provides an additional prognostic parameter as presence of fibrosis is associated with a significantly shorter progression-free survival (*p* = 0.001), splenomegaly (*p* = 0.016), and increased megakaryocytes (*p* = 0.04).

Presence of fibrosis had a negative impact in patients regardless of the blast-based categories (CMML-1 and CMML-2), degree of monocytosis, or severity of cytopenias, which are important factors in current prognostication tools [4, 10]. Previous studies indicated that patients with the proliferative variant of CMML, a WBC-based sub-category, have worse prognosis than those with the dysplastic variant [29]. However, we found no association between presence of fibrosis and these two WBC-based CMML subtypes. Splenomegaly has been reported to be more frequent in proliferative CMML based on earlier studies [30, 31], and we find that presence of fibrosis is associated with splenomegaly. However, the absence of an association between fibrosis and mean WBC, suggests biological independence of these phenomena. Due to the retrospective nature of this study, material is not available to evaluate for *RAS* mutations, which have been associated with the proliferative CMML subtype [29]. The association of CMML-F with increased BM megakaryocytes and splenomegaly may be explained by megakaryocyte role in mediating bone marrow fibrosis possibly by releasing transforming growth factor beta 1 (TGF-β1). Increased fibrosis can ultimately result in extramedullary hematopoiesis that commonly involves the spleen [32].

Splenomegaly is not uncommon in CMML with estimated frequency ranging between 17%–39% [30, 31, 33, 34] and is typically characterized by the leukemic infiltration of the red pulp by immature myelomonocytic cells and granulocytes [1]. Evaluation of splenectomy specimens from CMML patients performed to relieve thrombocytopenia have also identified foamy histiocytes and macrophages, trilineage extramedullary hematopoiesis [35], and tumoral proliferations of plasmacytoid dendritic cell nodules [36, 37].

The incidence of BM fibrosis in our cohort of CMML patients is 54%, and is seemingly higher than the previously reported incidence of 30% in the literature [18]. However, the prior work by Maschek and coworkers cannot be directly compared as it was published before the 2008 WHO classification and prior to the incorporation of the European BM Fibrosis consensus criteria. Furthermore, to our knowledge, our study is the only one that has focused specifically on CMML and fibrosis in the English literature. Moderate and severe fibrosis is rare in our cohort, as it is in the literature, where this finding has been occasionally described without addressing its incidence [38].

Our study also highlights the reproducibility of the WHO semiquantitative “MF” grading system since we found 97% concordance when 33 cases were re-assessed for fibrosis and compared to the original assessment. In the single case where the grade was changed (from original borderline grade of MF 1-2 downgraded to MF 1), this change made no impact on the patient group assignment since our study design specifically discriminated for presence or absence of fibrosis, and this patient remained in the same category. Thus, we feel that determination of presence (MF1+) or absence of fibrosis (MF 0) is a more robust assessment with less inter-observer reproducibility than discerning between MF1 and MF2 [39].

A limitation of our study is the lack of complete molecular profiling of the cases in our cohort, since most patients included in the study pre-date the more routine use of gene panels available currently. Given the retrospective study design, archival material suitable for molecular analysis is not available on our patients. Furthermore, combining data from two large referral centers highlights the variability in the typical work up at the time of diagnosis, since only a minority of patients had testing by a next-generation sequencing panel for common myeloid-associated somatic mutations. The presence of *JAK2* exon 12 and exon 14 mutations was evaluated for a large proportion of our patients; however, no correlation between fibrosis and a *JAK2* mutation was observed in our study. Mutations in *MPL* and *CALR*, typically associated with MPNs, were not identified among the small subset of patients tested, consistent with findings of the published molecular profiling studies of CMML [13]. It is important to note, that monocytosis has been associated with disease progression in several subtypes of MPN, specifically PMF and PV [28, 40]; however, none of our cases had clinical or pathologic features suggestive of this etiology. The heterogeneity of diagnostic workup at time of the initial diagnostic assessment similarly precludes us from being able to evaluate the immunophenotypic characteristics of the neoplastic monocytic cells by flow cytometry in these two patient groups. Larger studies, including evaluation by flow cytometry and complete molecular mutational characterization are needed to further explore our observations and evaluate our findings. In addition, larger studies are needed that systematically and prospectively incorporate BM fibrosis together with other prognostication tools.

In summary, the goal of this study was to characterize CMML patients with and without BM fibrosis and perform a comparison of their clinicopathologic features and outcomes. Our findings indicate that CMML patients with any BM fibrosis have increased megakaryocytes, more frequent splenomegaly, and a significantly shorter progression-free survival compared to CMML patients without any BM fibrosis. Based on these results, our study demonstrates the importance of assessing BM fibrosis in CMML. Similar to MDS, the presence of BM fibrosis may identify a distinct subgroup of CMML patients characterized by more aggressive behavior and earlier requirement for therapeutic intervention.

## MATERIALS AND METHODS

### Patients

We retrospectively identified in the pathology databases all consecutive patients with a diagnosis of CMML at two large referral medical centers (New York Presbyterian -Weill Cornell Medicine and Memorial Sloan Kettering Cancer Center) during an 8-year time interval (April 2007 through March 2015). The inclusion and exclusion criteria were as follows: 1) All cases met the diagnostic criteria for CMML according to the 2008 WHO criteria [1] and the diagnosis was further confirmed in conjunction with clinical follow-up data; 2) bone marrow biopsy specimens at the time of the initial diagnosis of CMML were assessed for fibrosis or were available for retrospective assessment of fibrosis; and 3) patients with another concurrent hematological neoplasm (e.g. primary myelofibrosis, mastocytosis, chronic lymphocytic leukemia, etc), post-treatment bone marrow examinations, or presentation in progressed phase (AML or leukemia cutis) were excluded. The study was approved by the institutional review boards at the participating institutions.

### Bone marrow assessment

**Figure 2:**
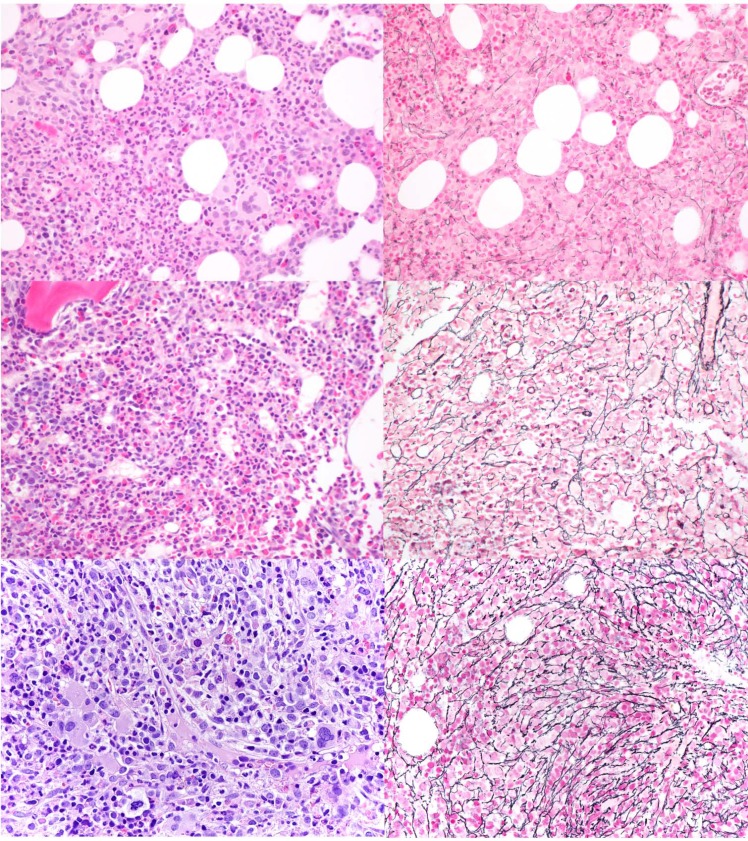
Representative BM biopsies of patients with CMML and various grades of BM fibrosis H&E (left) and the corresponding reticulin stain (right) at 400X in patients with CMML and no fibrosis (MF-0, top), mild fibrosis (MF-1, middle), and moderate fibrosis (MF-2, bottom).

All bone marrow biopsies were fixed in Bouin’s solution and acid decalcified. Hematoxylin and eosin sections were stained for morphologic review. Wright-Giemsa stained bone marrow aspirates were also reviewed. Reticulin stains were performed on all bone marrow biopsy specimens included in the study and were reviewed at each original institution at time of the initial interpretation. Bone marrow fibrosis was graded according to the WHO semiquantitative “MF” grading system, which is based on the European Bone Marrow Fibrosis Network criteria as MF 0, 1, 2, and 3 [19], demonstrated in Figure 2. In a few cases with heterogeneous reticulin deposition, fibrosis was assessed based on the more prevalent pattern. To confirm consistency in grading of fibrosis, a subset of cases (33/83, 40%) was re-reviewed without blinding to the initial interpretation with 97% (32/33) concordance with fibrosis grading at the initial diagnostic assessment (including MF-0: 7, MF-1: 17, MF-2: 7, and MF-3: 2). In a single case the grade was changed from the original borderline grade of MF1-2 downgraded to MF-1, and this change did not affect the subsequent comparison. Cases of CMML lacking bone marrow fibrosis (MF-0) were compared with cases of CMML with at least grade MF-1 fibrosis (MF1+). Morphologic review also included quantification of the marrow cellularity estimated after excluding background fibrosis. Blasts/ blast equivalents and monocytes were enumerated by standard practice as the percentage of total bone marrow nucleated cells on the aspirate, and these data were extracted from the pathology reports. If the aspirate was hemodilute or inadequate, a CD34 immunohistochemical stain was performed on the core biopsy for blast estimation after excluding endothelial cells (1/38 (3%) in the MF-0 and 4/45 (9%) in the MF1+ groups). Semi-qualitative assessment of megakaryocytes was performed on the BM biopsy and categorized as increased, decreased, or adequate. An average of 1–5 megakaryocytes per 40X field was defined as adequate [41, 42], while decreased and increased megakaryopoiesis corresponded to approximately < 1 and > 6 per 40X field, respectively.

### Laboratory data

Complete blood counts [hemoglobin (Hg), white blood cell (WBC) count, mean corpuscular volume (MCV), platelet count (Plt), and absolute monocyte count] concurrent with the initial BM sampling were recorded. The WBC count was used to sub-classify patients as belonging to the “proliferative” (WBC ≥ 13 × 10^9^/L) or “dysplastic” (WBC < 13 × 10^9^/L) CMML types [5].

### Molecular testing

DNA was extracted from peripheral blood or bone marrow samples. *JAK2* V617F mutation was assessed in the majority of cases (36) using amplification refractory mutation system-PCR assay [43] designed to amplify a segment of *JAK2* encompassing the codon for amino acid 617. In a minority of cases (12) *JAK2*, *MPL* exon 10, and *CALR* exon 9 gene mutations were evaluated as part of larger gene panels by a next-generation sequencing-based custom-designed assay using the Illumina MiSeq platform.

### Cytogenetic studies

Cytogenetic studies were performed on unstimulated overnight BM cultures and reported according to the 2009 International System for Human Cytogenetic Nomenclature [44]. When possible, a total of 20 G-banded metaphases were evaluated. Cytogenetic risk stratification was assigned as previously described [10]: low risk including normal karyotype and isolated loss of Y; high risk, including complex karyotype [≥ 3 abnormalities], trisomy 8, and abnormalities of chromosome 7; and intermediate risk, including all other abnormalities.

### Disease progression evaluation

Follow-up BM biopsies were evaluated as part of routine clinical monitoring. Disease progression on follow-up biopsy was defined as increase in blasts resulting in a change of the blast-based category, evolution to overt AML, or tissue infiltration by blasts. Treatment with and timing of a hematopoietic stem cell transplant were recorded. Presence of splenomegaly based on imaging or physical exam at diagnosis or during follow-up was recorded.

### Statistical analyses

Statistical data analysis was performed using R software for Windows (version 3.3.1). For continuous variables, data were reported as means and ranges and compared by the Mann–Whitney *U*-test. Fisher’s exact test or χ^2^ were used for categorical comparisons, as appropriate. Overall survival and time to disease progression were estimated by the Kaplan–Meier method and log-rank test. Patients who received stem cell transplant were censored at the time of transplant. All *P* values were two-tailed and were considered significant when < 0.05.
